# CariesCare International adapted for the pandemic in children: Caries OUT multicentre single-group interventional study protocol

**DOI:** 10.1186/s12903-021-01674-1

**Published:** 2021-07-01

**Authors:** Stefania Martignon, Andrea Cortes, Gail V. A. Douglas, J. Timothy Newton, Nigel B. Pitts, Viviana Avila, Margarita Usuga-Vacca, Luis F. Gamboa, Christopher Deery, Ninoska Abreu-Placeres, Clarisa Bonifacio, Mariana M. Braga, Fabiana Carletto-Körber, Patricia Castro, María P. Cerezo, Nathaly Chavarría, Olga L. Cifuentes, Beatriz Echeverri, Sofía Jácome-Liévano, Irina Kuzmina, J. Sebastián Lara, David Manton, E. Angeles Martínez-Mier, Paulo Melo, Michèle Muller-Bolla, Emilia Ochoa, Jesús R. Osorio, Ketty Ramos, Angie F. Sanabria, Johanna Sanjuán, Magdalena San-Martín, Aldo Squassi, A. Karina Velasco, Rita Villena, Andrea Ferreira Zandona, Edgar O. Beltrán

**Affiliations:** 1 UNICA - Caries Research Unit, Research Department, Universidad El Bosque, Av. Cra. 9 No. 131 A – 02, 110121 Bogotá, Colombia; 2 Dental Public Health, Leeds Dental Institute, University of Leeds, Leeds, UK; 3 Dental Innovation and Impact, Faculty of Dentistry, Oral and Craniofacial Sciences, King’s College London, London, UK; 4 School of Clinical Dentistry, The University of Sheffield, Sheffield, UK; 5 Biomaterials and Dentistry Research Center (CIBO-UNIBE), Academic Research Department, Universidad Iberoamericana UNIBE, Santo Domingo, Dominican Republic; 6 Department of Pediatric Dentistry, Academic Center for Dentistry Amsterdam, Amsterdam, The Netherlands; 7 Department of Paediatric Dentistry, School of Dentistry, University of São Paulo, São Paulo, Brazil; 8 Comprehensive Children and Adolescents Clinic, Paediatric Dentistry, Universidad Nacional de Córdoba, Córdoba, Argentina; 9 School of Dentistry, Corporación Universitaria Rafael Núñez, Cartagena, Colombia; 10 School of Dentistry, Universidad Autónoma de Manizales, Manizales, Colombia; 11 School of Dentistry, Universidad Cooperativa de Colombia, Envigado, Colombia; 12 Department of Preventive Dentistry, Moscow State University of Medicine and Dentistry, Moscow, Russia; 13 Department of Cariology, Operative Dentistry and Dental Public Health, Indiana University School of Dentistry, Indianapolis, IN USA; 14 Centrum Voor Tandheelkunde en Mondzorgkunde, UMCG, University of Groningen, Groningen, The Netherlands; 15 EpiUnit, Faculty of Dental Medicine, Institute of Public Health, University of Porto, Porto, Portugal; 16 Department of Paediatric Dentistry, Côte D’Azur University, Nice, France; 17 Viva 1A IPS Health Provider, Barranquilla, Colombia; 18 School of Dentistry, Universidad de Cartagena, Cartagena, Colombia; 19 Paedriatric Dentistry Department, Fundación Universitaria de Colegios de Colombia (UNICOC), Bogotá, Colombia; 20 School of Dentistry, Universidad Católica de Uruguay, Montevideo, Uruguay; 21 School of Dentistry, Universidad de Buenos Aires, Buenos Aires, Argentina; 22 Paediatric Dentistry Department, Universidad San Martín de Porres, Lima, Peru; 23 Department of Comprehensive Care, School of Dental Medicine, Tufts University, Boston, MA USA

**Keywords:** Dental caries, Children, COVID-19, Dental care, Conservative care, Aerosols, Remote consultation, Outcome assessment, Multicenter study

## Abstract

**Background:**

Comprehensive caries care has shown effectiveness in controlling caries progression and improving health outcomes by controlling caries risk, preventing initial-caries lesions progression, and patient satisfaction. To date, the caries-progression control effectiveness of the patient-centred risk-based CariesCare International (CCI) system, derived from ICCMS™ for the practice (2019), remains unproven. With the onset of the COVID-19 pandemic a previously planned multi-centre RCT shifted to this “Caries OUT” study, aiming to assess in a single-intervention group in children, the caries-control effectiveness of CCI adapted for the pandemic with non-aerosols generating procedures (non-AGP) and reducing in-office time.

**Methods:**

In this 1-year multi-centre single-group interventional trial the adapted-CCI effectiveness will be assessed in one single group in terms of tooth-surface level caries progression control, and secondarily, individual-level caries progression control, children’s oral-health behaviour change, parents’ and dentists’ process acceptability, and costs exploration. A sample size of 258 3–5 and 6–8 years old patients was calculated after removing half from the previous RCT, allowing for a 25% dropout, including generally health children (27 per centre). The single-group intervention will be the adapted-CCI 4D-cycle caries care, with non-AGP and reduced in-office appointments’ time. A trained examiner per centre will conduct examinations at baseline, at 5–5.5 months (3 months after basic management), 8.5 and 12 months, assessing the child’s CCI caries risk and oral-health behaviour, visually staging and assessing caries-lesions severity and activity without air-drying (ICDAS-merged Epi); fillings/sealants; missing/dental-sepsis teeth, and tooth symptoms, synthetizing together with parent and external-trained dental practitioner (DP) the patient- and tooth-surface level diagnoses and personalised care plan. DP will deliver the adapted-CCI caries care. Parents’ and dentists’ process acceptability will be assessed via Treatment-Evaluation-Inventory questionnaires, and costs in terms of number of appointments and activities. Twenty-one centres in 13 countries will participate.

**Discussion:**

The results of Caries OUT adapted for the pandemic will provide clinical data that could help support shifting the caries care in children towards individualised oral-health behaviour improvement and tooth-preserving care, improving health outcomes, and explore if the caries progression can be controlled during the pandemic by conducting non-AGP and reducing in-office time.

*Trial registration*: Retrospectively-registered-ClinicalTrials.gov-NCT04666597-07/12/2020: https://register.clinicaltrials.gov/prs/app/action/SelectProtocol?sid=S000AGM4&selectaction=Edit&uid=U00019IE&ts=2&cx=uwje3h. Protocol-version 2: 27/01/2021.

**Supplementary Information:**

The online version contains supplementary material available at 10.1186/s12903-021-01674-1.

## Background

Caries is a highly prevalent disease that has a global impact on health and well-being that is highly ranked. Untreated caries has been recently reported as the most prevalent global-burden-disease (GBD) condition, with an age-standardised prevalence of 34% in permanent teeth and 7.8% in primary teeth [1]. Traditional approaches to the assessment and management of dental caries were founded on a dichotomous determination of disease, and reparative approaches. As understanding of the disease process has developed alongside novel approaches to prevention and management, modern approaches emphasise caries as a process of demineralisation of the tooth surface, which can be reversed in its earliest stages, as well as the importance of prevention of the disease process [2, 3].

Though clinical studies have found that caries management systems aimed at preventing and controlling caries at the individual- and tooth-surface level, can achieve successful health outcomes and patient satisfaction with potential long-term cost-effectiveness [4–7], capability, opportunity and motivational barriers, amongst others, have been reported to affect the adoption of best clinical practice behaviors [8]. While the recently launched caries care system, CariesCare International (CCI) [9] was designed to help overcome these, its effectiveness in the control of caries progression has not yet been demonstrated.

CariesCare International was developed in 2019 as a health outcomes-focused patient-centered risk-based approach to caries management designed for the dental practice [9]. Its practice-friendly consensus guide, derived from the International Caries Classification and Management System (ICCMS™) [11, 11], promotes best practice as informed by the best available evidence to control the caries process and maintain oral health in their patients it advocates the ‘4D cycle’. This include: 1D: Determine Caries Risk; 2D: Detect caries lesions, stage their severity and assess their activity status; 3D: Decide a personalized care plan, and 4D: Do the preventive and tooth-preserving care, which includes risk-appropriate preventive care, control of initial non-cavitated lesions, and conservative restorative treatment of deep dentinal and cavitated caries lesions.

At the beginning of 2019, the Caries OUT collaboration were ready to start a 12-month multicentre pragmatic randomized clinical trial (RCT) in schoolchildren to compare the control of individual and tooth-level caries progression of the CCI system versus standard care. At that point, the collaboration had (1) achieved ethical approval via the leading centre, (2) recruited a number of clinical institutions, (3) obtained a partial funding from the IADR Regional Development Program for the Latin American Region (IADR RDP LAR), (4) prepared training material, and (5) planned training for researchers to start the study.

On March 11, 2020, the World Health Organization declared the global spread of coronavirus disease (COVID-19) a pandemic [12]. SARS-CoV-2 is a respiratory airborne virus that may be transmitted through dental procedures producing aerosols, either because virus remains suspended in the air or through contamination of inanimate surfaces. Aerosol generating dental procedures (AGPs) therefore are considered a potential source of infection for susceptible individuals [13, 14]. Consequently, elective dental procedures were stopped in the USA in March 2019 [15] and many other locations worldwide. This also led to the cancellation of many clinical dental studies, mainly randomized controlled trials, including the Caries OUT study as previously conceived.

**Fig. 1:**
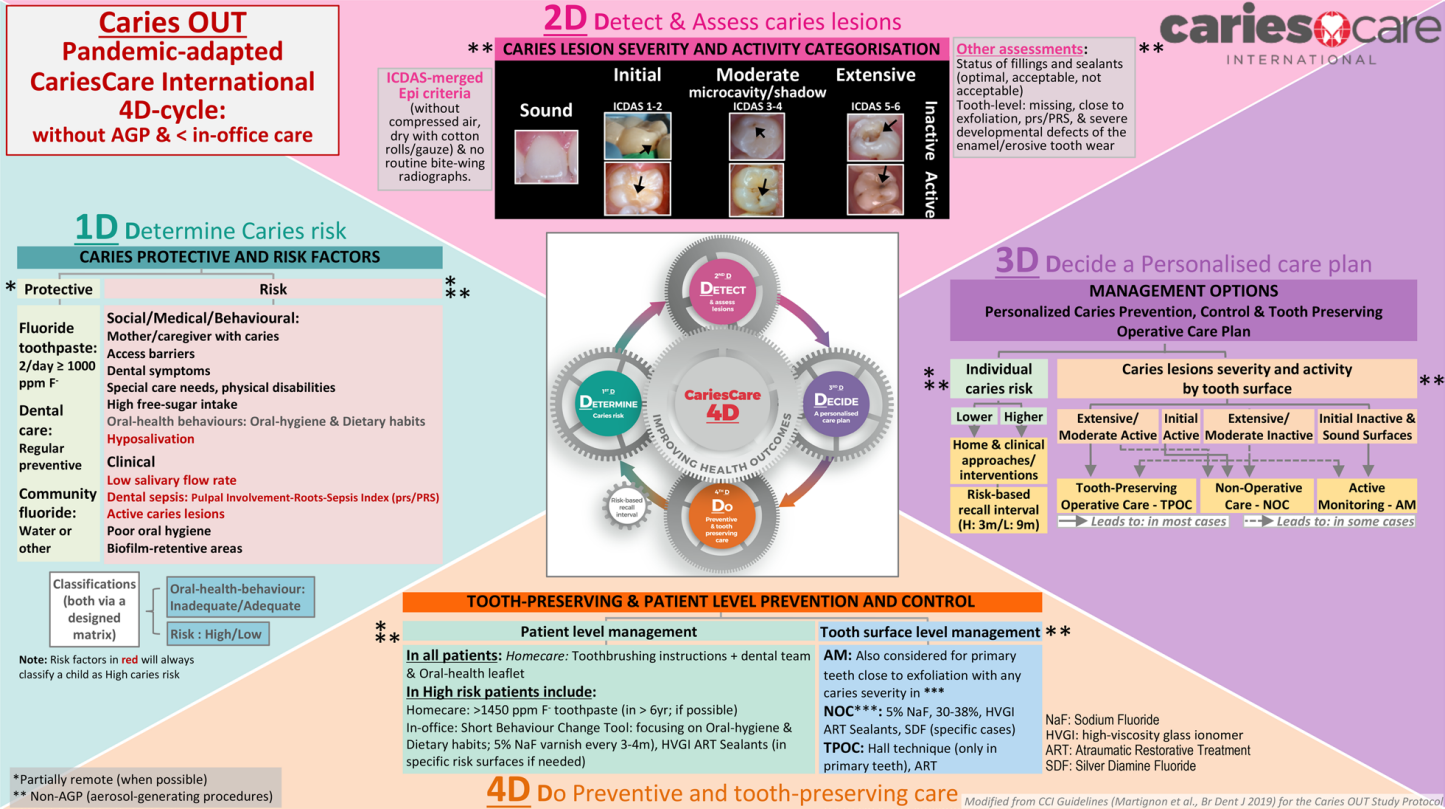
Caries OUT Pandemic-adapted CCI 4D-cycle

However, since May 2019 the Caries OUT collaboration have discussed how meaningful research in the current restricted clinical environment might still be conducted to inform caries management in children. This was felt to be particularly important given restrictions in current dental care which may contribute to an increase in the caries burden within the child population, especially in countries with higher health inequalities [16]. The collaboration worked to develop an adapted CCI Caries OUT single-group study, which would be viable without placing participants at increased risk of SARS-CoV-2 transmission. Amendments to the previous protocol includes managing caries in children with a 4D-cycle CCI adapted for the pandemic era, without AGPs [16–22], and reducing in-office time/appointments [16, 18, 19, 23] (Fig. 1). Processes during caries management which do not contribute to generating potentially contaminated droplets or aerosols, amongst other things include: air-drying only with cotton rolls/gauze for the visual detection (2D), isolating only with cotton rolls, cleaning the tooth surfaces only with toothbrushing by child/parent before the appointment and in the clinic with cotton rolls/gauze (2D and 4D). Avoidance of operative care and managing caries risk include the following non-operative care (NOC) options (4D): 5% NaF varnish, 30% silver diamine fluoride (SDF), high-viscosity glass ionomer atraumatic-restorative-treatment (ART) sealants [9, 16–22, 24]. For cavitated caries lesions, either NOC or tooth-preserving operative care (TPOC) with Hall technique (for primary teeth) and ART are options (4D) [9, 16–22, 24–27]. Teledentistry text messages or videocalls can contribute to reducing in-office time/appointments [16, 18, 19, 23, 28]; these approaches can be used for the assessment of the caries risk (1D) as well as for its management via advising on home-care approaches (4D), and with oral-health behaviour change strategies focusing on toothbrushing and dietary habits [9, 29–34].

Thus, the Caries OUT collaboration, with agreement from each of the institutions and funding bodies, proposed a revised research plan to conduct a clinical study with only one arm assessing a pandemic-adapted CCI system. The immediate benefits of such a study may be the training and empowerment of researchers/staff/dental practitioners to offer safe and appropriate caries care to children during the current COVID-19 epidemic and thus contribute to reducing the burden of caries which might otherwise increase. The aim of this 12-month multicentre single-group interventional study is to assess in children the caries-control effectiveness of a pandemic-adapted CCI protocol (non-AGP and reduced in-office appointments’ time), in terms of child-level and tooth-level control of caries progression; acceptability of care to parents and dentists; and change in children’s oral health behaviours.

## Methods/design

The design and report of this clinical trial protocol follows the Standard Protocol Items: Recommendations for Interventional Trials (SPIRIT) statement (Additional file 1). The study received approval from the Research Institutional Ethical Committee in Universidad El Bosque (PCI 2019-10718).

This is a 12-month multicentre single-group interventional study that aims at assessing in children the caries-control effectiveness of a pandemic-adapted CCI protocol (non-AGP and reduced in-office appointments’ time). The outcomes include primarily, tooth-level control of caries progression, and secondarily, child-level control of caries progression and caries risk; parents’ and dentists’ acceptability of care, and improvement in children’s oral health behaviours. Data will be collected in the 21 centres that agreed to participate, which are located in Argentina [2], Brazil [1], Colombia [7], Dominican Republic [1], France [1], Mexico [1], Perú [1], Portugal [1], Russia [1], The Netherlands [1], United Kingdom [1], United States [2], and Uruguay [1].

**Fig. 2:**
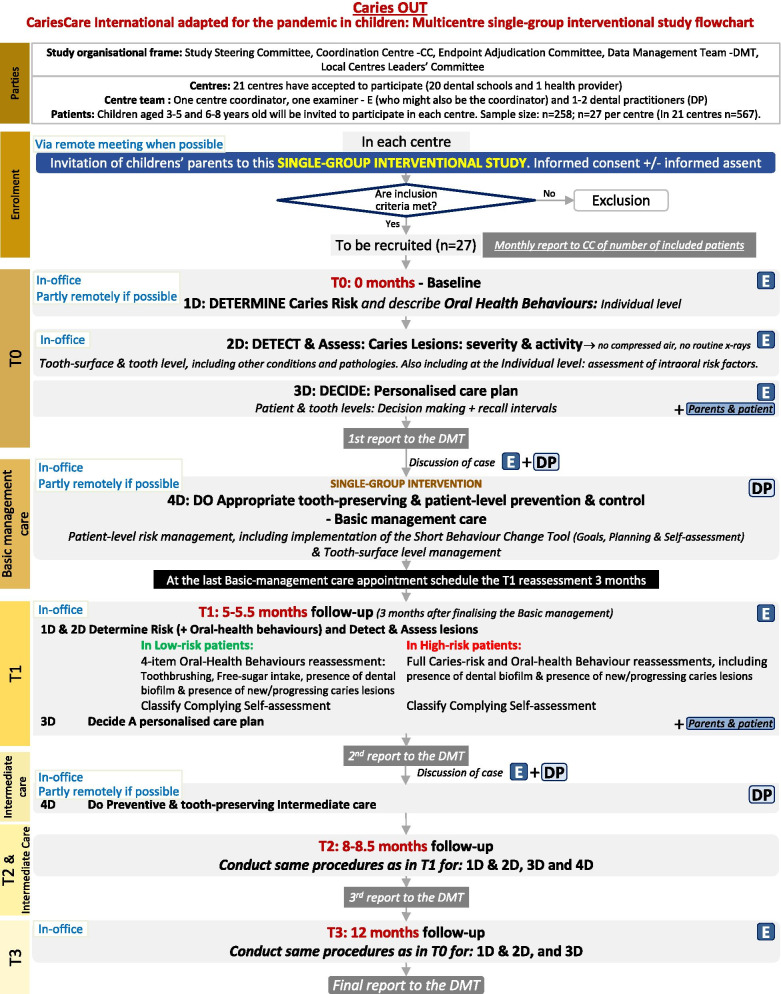
Caries OUT Pandemic-adapted CCI study flowchart

The study phases are based on the CCI 4D cycle (Fig. 1) and include: (1) a baseline examination (T0), corresponding to CCI steps 1D, 2D and 3D to be conducted by the examiner (E); (2) the basic management and intermediate care (CCI step 4D) to be conducted by the dental practitioner/s (DP); (3) two follow-up assessments during the 12-month care period: T1, at 5–5.5 months (3 months after end of basic management care) (E); T2, at 8.5 months, and (4) a final re-assessment at 12 months (T3) (E). The study flowchart is shown in Fig. 2.

### Setting, participants and recruitment

Each centre will define where they will conduct the local single-group interventional study. This can be either in a dental school clinic, in a dental schools’ community/hospital/school children’s clinics, or if these are not available due to the pandemic, in researchers’ private practices. Invitations will be sent to these clinics and upon interest to participate, the centre coordinator will explain the study, its purposes and procedures. When and if the clinics’ authorities accept to take part, corresponding invitation and information about the study will be sent to the participants’ parents (and children), together with written consent forms (Additional file 2). Written assent forms will be included in countries where it is required and also depending on the age requirement (e.g. in Colombia for children over 6 years) (Additional file 3).

The inclusion criteria are children: (1) aged 3–5 and 6–8 years old; (2) generally healthy, and (3) have parental consent and children’s assent (if needed). The exclusion criteria include children who: (1) have major systemic diseases or major mental/physical disability; (2) their family have plans to move during the study timeline, and (3) wear orthodontic appliances. The strategies to reach the target sample size at enrolment, include the motivation of parents through the invitation, explaining the advantages of having their children receiving a caries patient-centred care and risk-based prevention, and that they will be receiving printed didactic aids to improve the child’s oral health behaviours. Children who attend a different dental practice for care during the study period will be excluded.

Written consent forms signed by parents/carers and written assent forms (when these applies) signed by children will be collected from children interested in participating before the dental examination and treatment take place and subjects will be coded to keep confidentiality.

### Baseline and follow-up oral examinations

Baseline (T0) and follow-up examinations (T1, T2, T3) will be conducted in each centre at the appointed dental clinic/s (including remote acquiring of interview data if possible and when applicable) by an examiner (E) previously trained in the ICDAS visual caries criteria [35] and in the CCI steps 1D, 2D and 3D [9]. The caries management (4D) will be delivered by external trained dental practitioner/s (DP). These steps are detailed in Fig. 2.

For 1D, remote combined with in-office assessment will be conducted with parents, including social/medical/behavioural risk factors (6 items); protective factors (3 items), and oral hygiene (3 items) and dietary (5 items) oral-health behaviours [3, 9, 10, 29, 31–34, 36]. Clinical risk factors (5 items) will be assessed in the dental clinic with the children, as 2D assessments. After assessing dental biofilm (Silness and Löe modified, [37]) , the other clinical assessments are conducted after toothbrushing, with the aid of a WHO probe and drying tooth surfaces and/or removing any dental biofilm only with cotton rolls/gauze (without using compressed air/water). Assessments at the tooth-surface level include: (1) caries lesions staging and activity assessment using the visual ICDAS-merged Epi criteria (Sound, Initial, Moderate microcavity/shadow, Extensive) active/inactive [35], and (2) presence and status of fillings (Optimal, Acceptable or Not acceptable) and sealants (Optimal or Not acceptable), modified from Cvar and Ryge [38], and at the tooth-level: (1) missing due to caries; (2) tooth close to exfoliation, and (3) dental sepsis as a clinical consequence of untreated caries, with the PuIpal Involvement-Roots-Sepsis Index (prs/PRS) modified from the PUFA Index [39] with/without toothache. Routine bite-wing radiographs are not included and only used in specific cases based on clinical need, not as part of the study protocol. For 3D, after synthesis of information from steps 1D and 2D the examiner conducts the personalised care plan, jointly with the child’s parent and the external DP. At the individual-level, a designed matrix based on CCI [9] weighs up the risk and protective factors against each other, classifying the patient’s caries risk into low or high, with a respective 9-month or 3-month risk recall interval. Another matrix, taking into account the COM-B behaviour model [29] weighs up the adequate vs. inadequate oral hygiene and dietary habits behaviours against each other, classifying the patient’s oral-health behaviour into adequate, inadequate or very inadequate. The individual-level risk management decisions will include homecare and clinical approaches/interventions, according to CCI and adapted for the pandemic (with non-AGP and including remote appointments when possible), including implementing a short behaviour-change tool. At the tooth-surface level, according to the synthesis of the clinical assessments defined in 2D, the type of care is defined per surface with non-AGP as: none, active monitoring (AM), non-operative care (NOC), tooth-preserving operative care (TPOC), and at the tooth-level as endodontics or extraction [9, 9].

T0 and T3 include full 1D, 2D and 3D assessments. For intermediate T1 and T2 follow-ups, in 1D, all receive full oral-health behaviour reassessment, while risk is fully assessed only in patients previously classified as high-risk; in low-risk, only four risk/protective factors are reassessed (twice-a-day toothbrusing with ≥ 1000 ppm F, daily free-sugar intake above 50 g, dental biofilm and presence of new caries lesions). For 2D, all patients are clinically examined only for the latter. Subsequent 3D is derived. If in T1 or T2 the patient’s risk gets classified opposite to their previous assessment, both the recall interval and the reassessment are adjusted accordingly.

Parents’ and dentists’ acceptance of dental care will be assessed by an external researcher at T1, using designed TEI questionnaires (modified from Newton & Sturmey [40]).

Costs will be assessed in terms of duration and number of appointments regarding the type of care received (assessment/reassessment; individual level: risk/behavioral management, recall; tooth-surface level: NOC, TPOC;) and taking into account if the activity has been conducted solely by the examiner/CD, solely by a hygienist or both together, and country-level economic variables will be described [41–43].

Dropout criteria of a child from the study will include: (1) voluntary withdrawal from the trial by the patient/parents; (2) not attending the reassessments and in-office/remote appointments after three phone/message reminders.

### Interventions

**Fig. 3:**
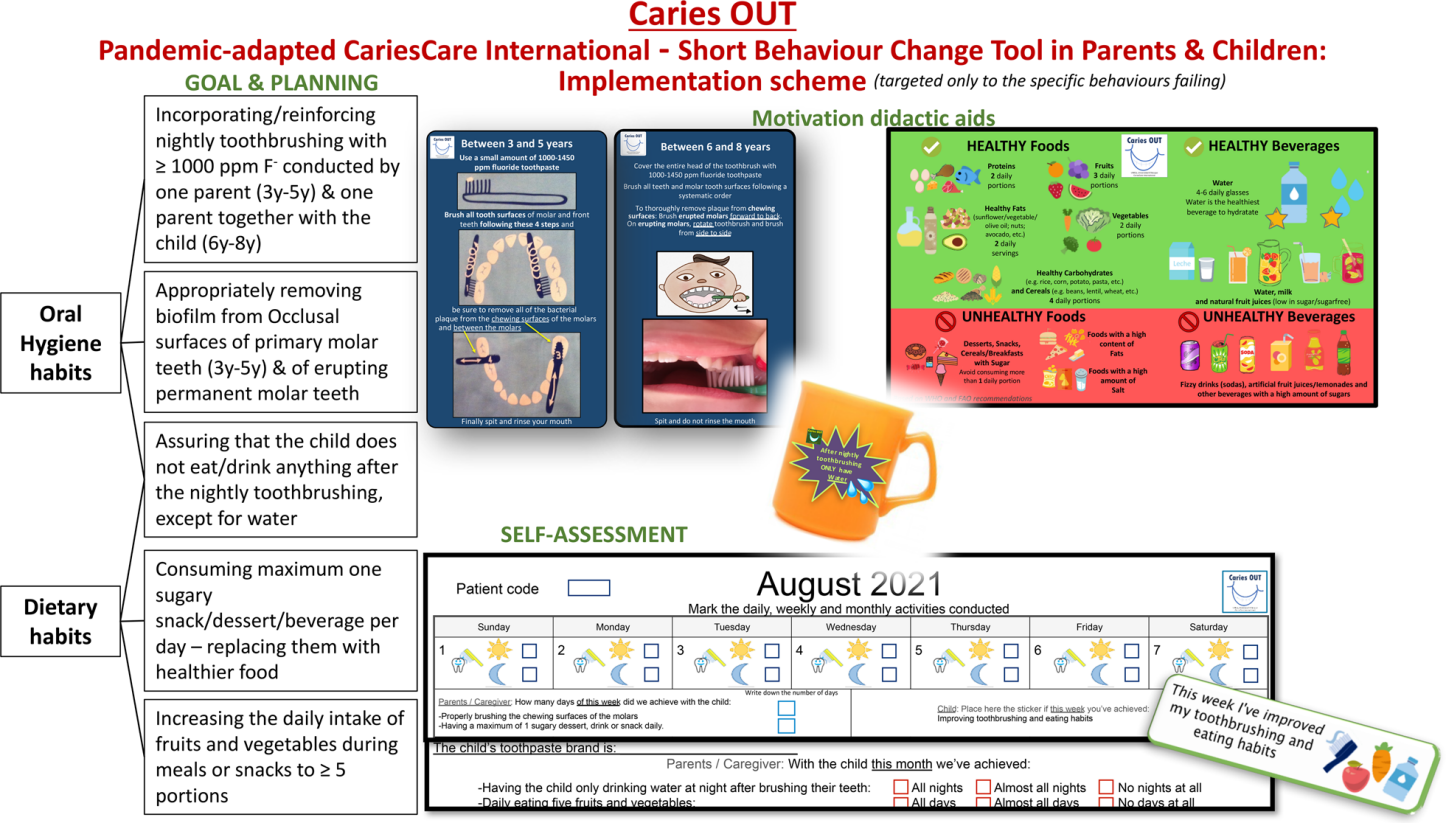
Caries OUT short-behaviour-change tool implementation in parents and children

The interventions of this single-group study correspond to the 4D, as consequent individual- and tooth-level management approaches/interventions, to be implemented by the external DP, when possible with remote care and only with non-AGP. At the individual level (Fig. 1), homecare approaches include for all patients, instructions on twice-a-day toothbrushing with ≥ 1000 ppm F, and for high-risk patients, increasing the toothpaste’s fluoride concentration and providing general oral-health improvement information; clinical approaches/interventions include 5% NaF varnish at basic management, T1 and T2; if it applies, high-viscosity glass ionomer ART sealants in Occlusal surfaces (under non-AGP conditions), and motivational engagement of patients and parents through the study-specifically designed Short Behaviour Change Tool (SBCT) [29]. The implementation of the SBCT consists of discussing with the parent each behaviour previously assessed as inadequate (Fig. 3). First, setting a goal to be accomplished (e.g. “Do you think you could incorporate at any point during the child’s evening activities toothbrushing using fluoride toothpaste every night?”); then, planning together how best to achieve it and supporting it with a didactic aid (e.g. a tooth-brushing adhesive instructive diagram for the bathroom mirror), and plan for self-monitoring (e.g. calendar to mark the daily activity). Low-risk patients receive the didactic aids but no behaviour change is conducted.

Interventions at the tooth-surface level include only non-AGP (4D) (Fig. 1) and are detailed in Table 1 for primary teeth and in Table 2 for permanent teeth, depending on the caries lesions’ severity and activity status, with AM, NOC and TPOC options.

**Table 1:** Caries OUT pandemic-adapted CCI-4D interventions at the tooth-surface level with non-AGP for primary dentition

	4D at the surface level: primary dentition		
ICDAS-merged Epi score	Caries OUT modified-CCI—care options		
Initial caries			
Inactive	AM		
Active	NOC	*Fossae and fissure:*	*Smooth surfaces:*
		*Option 1*: High-viscosity Glass Ionomer ART sealant + 5% NaF (every 3–6 m)	SDF 30–38% (every 6 m)/5% NaF (every 3 m)
		*Option 2:* 30–38% SDF (every 6 m)/5% NaF (every 3 m)	
		*In teeth close to exfoliation*: option 2 (1 time)	
Moderate caries microcavity/shadow			
Inactive	AM	After clinical judgement and *in teeth close to exfoliation*	
	NOC	*Fossae and fissure:*	*Smooth surfaces:*
		*Option 1:* High-viscosity Glass Ionomer ART sealant	*Option 1:* 30–38% SDF (1 time)/5% NaF (1 time)
		*Option 2:* 30–38% SDF (1 time)/5% NaF (1 time)	*Option 2:* High-viscosity Glass Ionomer ART sealant
Active	NOC	*Option 1*: High-viscosity Glass Ionomer ART sealant + 5% NaF (every 3–6 m)	
		*Option 2:* 30–38% SDF (every 6 m)/5% NaF (every 3 m)	
		*In teeth close to exfoliation* option 2 (1 time)	
	TPOC	*If there is a more advanced Moderate lesion and in a High-risk patient*: ART/Hall Technique	
When considered it necessary have a bitewing radiograph taken to assess the depth of the radiolucency and correlate it with the likelihood of dentine infection (in the middle dentine third). Register radiographic score in 2D, and combine it with the clinical score and the individual caries risk to decide in 3D the specific tooth-surface care plan (NOC/TPOC)			
Extensive caries			
Inactive	AM	After clinical judgement and *in teeth close to exfoliation*	
	NOC	High-viscosity Glass Ionomer ART sealant/30–38% SDF (1 time)/5% NaF (1 time)	
Active	NOC	*Option 1*: High-viscosity Glass Ionomer ART sealant + 5% NaF (every 3–6 m)	
		*Option 2:* 30–38% SDF (every 6 m)/5% NaF (every 3 m)	
	TPOC	*Molar teeth*	*Anterior teeth:*
		*Option 1*: Hall Technique	*Option 1*: ART
		*Option 2*: ART	*Option 2*: 30–38% SDF (every 6 m)
		*Option 3*: 30–38% SDF (every 6 m)	
		*In teeth close to exfoliation:* High-viscosity Glass Ionomer ART sealant + 5% NaF (1 time)/30–38% SDF (1 time)	
		*In deep cavities or reversible pulpitis (without other pulp symptomatology):* One-Step Excavation + ART	

If SDF is available in the centre and the parents or the child don’t accept to have it applied on anterior teeth, select an alternate option

In case of dental emergency/endodontic treatment need take an x-ray register the detection in 2D and 3D, and treat/refer, excluding that tooth

*AM* active monitoring, *NOC* non-operative care, *TPOC* tooth-preserving operative care, *ART* atraumatic restorative treatment, *NaF* sodium fluoride, *SDF* silver diamine fluoride

**Table 2:** Caries OUT pandemic-adapted CCI-4D interventions at the tooth-surface level with non-AGP for permanent dentition

	4D at the surface level: permanent dentition		
ICDAS-merged epi score	Caries OUT modified CCI—Care option		
Initial caries			
Inactive	AM		
Active	NOC	*Fossae and fissure:*	*Smooth surfaces:*
		*Option 1*: High-viscosity Glass Ionomer ART sealant + 5% NaF (every 3 m)	5% NaF (every 3 m)
		*Option 2:* 30–38% SDF (every 6 m)/5% NaF (every 3 m)	
Moderate caries microcavity/shadow			
Inactive	AM		
	NOC	*Fossae and fissure:*	*Smooth surfaces:*
		*Option 1:* High-viscosity Glass Ionomer ART sealant	*Option 1:* 5% NaF (1 time)
		*Option 2:* 30–38% SDF (1 time)/5% NaF (1 time)	*Option 2:* High-viscosity Glass Ionomer ART sealant
Active	NOC	*Option 1*: High-viscosity Glass Ionomer ART sealant + 5% NaF (every 3–6 m)	
		*Option 2:* 30–38% SDF (every 6 m) (only in molar teeth)/5% NaF (every 3 m)	
	TPOC	*If there is a more advanced Moderate lesion and in a High-risk patient*: ART	
When considered it necessary, have a bitewing radiograph taken to assess the depth of the radiolucency and correlate it with the likelihood of dentine infection (in the middle dentine third). Register radiographic score in 2D, and combine it with the clinical score and the individual caries risk to decide in 3D the specific tooth-surface care plan (NOC/TPOC)			
Extensive caries			
Inactive	AM		
	NOC	High-viscosity Glass Ionomer ART sealant/30–38% SDF (1 time)/5% NaF (1 time)	
Active	NOC	30–38% SDF (every 6 m)	
	TPOC	ART	
	*In deep cavities or reversible pulpitis (without other pulp symptomatology):* One-Step Excavation + careful ART		

In case of dental emergency/endodontic treatment need take an x-ray register the detection in 2D and 3D, and treat/refer, excluding that tooth

*AM* active monitoring, *NOC* non-operative care, *TPOC* tooth-preserving operative care, *ART* atraumatic restorative treatment, *NaF* sodium fluoride, *SDF* silver diamine fluoride

Interventions at both individual-and tooth surface level, will be conducted during the 12-month study period. After the 1-year follow-up (T3), no further interventions will be conducted for the study, but can be conducted separately if the centre decides to.

The strategies to improve adherence to interventions will include phone and text messages of next appointment reminders for parents and children; a remote communication channel with parents to answer questions and to motivate both the parents and the children to follow the study recommendations; reassuring the parents that in the practice the global SARS COV-2 biosafety guidelines including the use of PPE are being strictly followed. These strategies will also be used to promote participant retention and complete follow-up.

### Outcomes

#### Primary health outcome

- Mean number of tooth surfaces with avoidance of caries progression (ICDAS-merged Epi severity and/or activity).

#### Secondary health outcomes

- Proportion of subjects with avoidance of caries progression (ICDAS-merged Epi severity and/or activity).
- Proportion of subjects with avoidance of caries risk level increase/no control, and avoidance of extraction, pain, failure of the filling/sealant.
- Proportion of parents and dentists with high dental care process acceptability (measured with TEI).
- Proportion of children improving oral-health related behaviours.
- Description of dental care costs.

### Sample size and recruitment

The sample size was determined based on the sample size calculated for the previous randomized clinical trial, which was based on Curtis et al. [42]. We decided to use the mentioned study, even though it is on adults, as it is one of the very few available studies that deals with the management of caries using an updated system, similar to CariesCare International, taking into consideration both the care of caries lesions according to their severity and activity status, as well as of the individual caries risk. The results show differences in averages of surfaces with caries progression, between two preventive care schemes. The Whitehead sample size calculation formula was taken into account, with type-I error: 0.05, type II error: 10%, standard deviation 2.5, expected average of the first group 1.3 and expected average of the second group 2.1.

For the current study, as there is no control arm, with the leader team it was decided to include half of the previous RCT sample size. Thus, the sample size calculation of this single-group interventional study corresponded to 206 3–5 and 6–8 years old children, increasing to a total of 258 after including a 25% dropout. As the sample calculation per arm in each centre in the previous study corresponded to 20 participants, in this single-interventional study we are asking each centre to recruit 27 patients. These would correspond to 567 participants in total if the centres [21] finish the study with 27 participants each.

### Statistical analysis plan

All the baseline and follow-up the individual- and tooth-level data, the parents’ and dentists’ TEI data, and costs’ information will be digitally registered (after training) for each codified patient in each centre keeping data safely stored and with limited access, in a designed data base in Microsoft Excel (2010), that includes data quality assurance by validation of data. The data will be sent to the DMT at each time point. Data will be analysed by an independent statistician. All statistical tests will be two-tailed tests. The level of statistical significance for all two-sided tests will be set at 0.05. Parametric methods will be considered first. Data that do not meet or cannot be transformed to meet parametric assumptions will be analysed by non-parametric methods. Demographic and clinical features of the participants (centre, gender, age, caries risk, pain, filling status, prs/PRS); oral health behaviour, dental care acceptability and costs, as well as caries experience (DMFS and dmfs with the D component using ICDAS-merged Epi) [35] will be described using mean and standard deviation (SD) for quantitative variables and percentages for qualitative variables.

Regarding costs, the providers’ payment model of the centre will be described (Fee-for-service, Capitation, Salary-based and Pay-for-performance) [43]. For the description of costs, these will be converted to the United States Dollar (USD) under the average Market Representative Exchange Rate for the year 2021 (MRER-average).

ANOVA and Kruskal Wallis analyses will be used to compare the baseline characteristics between centres.

The caries risk control (subjects where risk was reduced from high risk or maintained in low risk), and the number of subjects with avoidance of extraction, pain, and failure of restorations/sealants, will be compared between baseline (T0) and 12-month follow-up (T3) examinations using χ^2^.

For the analyses at the tooth-surface level, we will exclude teeth that needed a treatment involving AGP (endodontic treatment, surgical extraction, etc.). Caries progression has been defined for this study in terms of a change from the decided and delivered tooth-surface level care (T0, after the 4D basic management) to the tooth-surface level status at the 12-month follow-up (T3) (Fig. 2), as follows: (1) from a sound surface to a caries lesion, a sealant, a filling, or a missing tooth; (2) from a sealant to a caries lesion, a filling, or a missing tooth; (3) to a more severe caries score and/or an active status (or remaining active), and (4) from a filling to a caries lesion or a missing tooth. The severity/activity caries progression will be firstly assessed through descriptive analyses (number of subjects/surfaces) and a comparison in the number of active lesions at 1 year versus baseline will be applied (Wilcoxon test or paired *t* test based on results of the Shapiro Wilk normality test). Multilevel logistic regression will also be used for the surface level data analyses, exploring the clustering of surfaces within individuals, to estimate the association among characteristics variables and the outcomes.

Parents’ treatment evaluation inventory: Comparisons of parents’ treatment acceptability will be conducted between participants classified in baseline as low vs. high risk using Independent *t* test.

Dentists’ treatment evaluation inventory: The dentists’ treatment acceptability will be described.

Oral-health related behaviour classification will be compared between assessments (T0, T1, T2, T3) and between high and low-risk patients using *t* test.

The level of significance considered for all tests will be 5%.

Analyses to handle protocol non-adherence and missing data: Data of patients who leave before the end of the study will be handled separately. A *t* test analysis will be conducted to compare the baseline mean number of surfaces with caries experience (ICDAS-merged Epi dmfs/DMFS) [35] of those children who left the study during the 1-year follow-up period with that of those who remained in the study. If they’ve left after T1 and or T2, their parents TEI will be also compared with that of those who remained, as well as the children’s oral-health behaviour classification, and the caries progression.

Oversight and monitoring: Throughout the study virtual meetings will be held to solve any adverse situation and to resolve any issues or concerns about the study protocols.

Adverse events and harms: There is a minimal risk of participating in this study, similar to that of routine dental care. All procedures involved have scientific support and are part of best clinical practices. Disposable materials and sterilized instruments will be used. Adverse events, if any, will be recorded and reported to the Coordinating Centre. Its management will follow dental clinics’ guidelines.

Auditing: The centres’ coordinators will be reporting any protocol deviation and updating periodically to the Endpoint Adjudication Committee. The Study Steering Committee will periodically audit the general conduction of the study. The Coordinator Centre will periodically audit the centres by assessing the data bases of a number of subjects independent from the centre dental team. The audit processes are independent from the sponsor.

Protocol amendments: According with the Research Institutional Ethical Committee in Universidad El Bosque, any modification to the project or to the approved forms must be submitted to them for its approval.

## Discussion

This is a multicentre single-group intervention study that will assess the caries-control effectiveness of CCI adapted for the pandemic outcomes in children. While the previous proposal for the study was a RCT which would compare CCI with standard care, the COVID-19 pandemic obliged a change of plan for this study to only one intervention group and to test an adapted CCI, without AGP and reduced in-office appointments’ time. Children have the right to receive an effective dental care even during the pandemic [16, 44, 45]. The CCI system is a comprehensive caries care system focused in improving oral health outcomes through a patient-centred risk-based friendly management for the practice [9]. The proposed care options have been recognised as effective for caries care [4–7, 24–27, 30, 33, 34, 36, 46] and most have been proposed as being suitable for this pandemic era [16–22], as have teledentistry and other patient’s remote communication means [16–18, 22, 23]. The short behaviour change tool helps in the management of caries risk by linking the patient as an active actor to improve oral health habits [29, 30]. Given the characteristics of the proposed adapted-CCI system, we will explore the effectiveness control of caries progression at both tooth-surface and individual-level, oral-health behaviour improvement, and parents’ and dentists’ process acceptability. If the results are positive, this will help to change the standard of care during the pandemic and beyond it. Results will be widely available to increase the translation and adoption of CCI in other countries, as well as contribute to the evidence in the use of non-AGP and remote appointments during the pandemics.

Compared to other childhood diseases, caries in both the permanent and the primary dentition is highly prevalent (1st and 12th, respectively) (1). Additionally, disease rates are higher in young children and schoolchildren in low- and middle-income populations. In deciding which age groups to study we considered 3–5 and 6–8 years old children to be important particularly due to changing diet and lifestyles, as well as the additional caries risk that the eruptive first permanent molar teeth pose [47, 48].

Taking into account that this study will only consider non-AGP procedures, the caries-progression outcome will be assessed with visual criteria by means of ICDAS-merged Epi, drying tooth-surfaces only with cotton/gauze. While some ICDAS initial lesions might be not detected, this modification of the criteria has been successfully used previously to assess caries progression in school settings, demonstrating its practicality and reliability [49].

While tooth-surface caries progression is the primary outcome, individual-caries-risk control, as well as oral health behaviour change and parents’ and dentists’ care acceptability will also be assessed. Oral-health behaviour change is also relevant within the context of increasing, e.g. oral-hygiene and dietary habits, to help in preventing caries [29]. On the other hand, the process acceptability of the adapted-CCI to both parents and dentists will contribute to understanding if the system is feasible [40].

The adapted-CCI homecare and in-office approaches and interventions are safe for clinical use and reduce the risk of SARS-Cov-2. Moreover, international and local biosafety and PPE considerations are in place, reducing ethical concerns for this study.

Finally, the fact that the implementation of this protocol has been planned to be conducted during the current pandemic situation, raises some challenges, on one hand, related to current ethical restrictions of the participating 21 centres, where the only possibility was to conduct the study as a single-group interventional study—losing the comparability with a control group; on the other hand, centres’ local feasibility, such as start, conduct and finish dates, under public health and governmental uncertainties. In this sense, the centres and researchers involved are putting a lot of effort in pursuing it.

## Supplementary Information

**Additional file 1**. Standard Protocol Items: Recommendations for Interventional Trials (SPIRIT) statement.

**Additional file 2**. Written consent form.

**Additional file 3**. Written assent form.

## Data Availability

Subjects will be coded in each site and data will be stored digitally by the (DMT) in UNICA at Universidad El Bosque to guarantee the subject’s confidentiality. The protocol, the datasets generated and/or analysed during the current study as well as the statistical analysis plan are available from the corresponding author on reasonable request. The child oral-health status at each follow-up examination will be informed verbally to each participant’s parents. The results of this study will be communicated by publications and presentations in international conferences and to the centres. Only the Steering Study Committee will have access to the final trial dataset, and previous contractual agreements with the centres will limit their access.

## Abbreviations

AGP: Aerosols Generation Procedures
AM: Active Monitoring
ART: Atraumatic Restorative Treatment
BM: Basic management
CCI: CariesCare International™
DP: Dental practitioner
ICCMS: International Caries Classification and Management System™
NOC: Non-Operative Care
Non-AGP: Non-Aerosol Generation Procedures
PPE: Personal Protection Equipment
RCT: Randomized controlled trial
SBCT: Short Behaviour Change Tool
TPOC: Tooth-Preserving Operative Care
T0: Baseline examination
T1: First reassessment
T2: Second reassessment
T3: Final reassessment
TEI: Treatment Evaluation Inventory
USD: United States Dollars
