# Supplementary material for: CariesCare International adapted for the pandemic in children: Caries OUT multicentre single-group interventional study protocol
Source: BMC Oral Health. 2021 Jul 1;21:329. doi: 10.1186/s12903-021-01674-1 (PMC8248759; doi:10.1186/s12903-021-01674-1)
Supplement: Supplementary file 2 — Additional file 2. Written consent form. [file 12903_2021_1674_MOESM2_ESM.pdf]

## RESEARCH TITLE: " Caries OUT: Multicenter Study in children with CariesCare International adapted for the COVID-19 pandemic".

## Informed Consent

### Part 1: Research Information.

- PROTOCOL NUMBER: PCI-2019-10718
- PRINCIPAL INVESTIGATOR NAME: Edgar Beltran.
- BACKGROUND

Dental caries is a disease of dental tissues occurring when oral hygiene habits are deficient and the consumption of sugars is not regulated (frequency and quantity).
Current guidelines for the understanding and management of caries classify this disease as preventable and detectable in early stages. Therefore, its management should start very early in childhood, based on the needs, lifestyle of each person to avoid future dental pain, dental loss and chewing problems of individuals.

In this study, we propose to evaluate a new caries management system called CariesCare International or simply CCI in which rotatory dental instruments will not be used because these instruments generate aerosols that could contain viral and bacterial pathogens that could cause infection - if they are not properly handled. The CCI management was approved in a consensus of experts at the national and international level. It allows identifying the probability that a person will develop caries and diagnose it early based on their needs and without using rotatory instruments. Rotatory instruments work slicing damaged dental structure quickly. Its use can be replaced by the use of other instruments similar to spoons that correspond to the same function of the rotatory structures.

Once this background is presented we are inviting you and your child to participate in this project. Once you agree to participate CCI will be used to take care of their child's oral health. The results will help us to see if CCI is effective in preventing tooth decay and in maintaining good oral health. It is also very important that during the management the child feels completely comfortable and you as a parent will also be satisfied with this. Therefore, at the end of the management you both will be invited to answer some questions.

WHAT DOES THIS INVESTIGATION AIM?
It aims to evaluate the result of applying the CCI strategy in the management of caries in children from 3 to 5 years old and from 6 to 8 years old, to avoid the presence / onset of caries lesions and to identify satisfaction in the CCI management. If you both agree to participate the child oral health will be assessed at 4 times: at the beginning of the study, after 3, 6 and 12 months. Additional telephone controls will be conducted. In addition, based on the oral exam appropriate treatments will be performed without the need to use aerosol-generating procedures. Together, these aspects will help us to identify problems in your oral health hygiene and habits.
WHAT DO I HAVE TO DO IF I PARTICIPATE IN THIS RESEARCH?
1) Before starting the investigation: you must know what the oral assessment will consist of, know about the generalities of the treatments that are handled under the CCI scheme. When you have no questions about this, you will be asked to sign this document if you agree to participate in the project, 2) During the research: you must guarantee the child's attendance to the appointments that are scheduled and that the child follows the instructions given by the researchers (the number of appointments will depend on what is given

UNIVERSIDAD EL BOSQUE

[illegible]

detect in the initial oral examination) and, 3) After the investigation is finished: you might guarantee the follow-up of the recommendations of the investigators and guarantee the attendance to the control appointments and / or attend the follow-up telephone calls and, 4. Respond telephone satisfaction surveys at the end of management.

HOW MANY PEOPLE WILL PARTICIPATE IN THIS RESEARCH?
420 children from 3 to 5 years old and/or from 6 to 8 years old. They are living in different cities and countries. In this institution 20 children will participate.

HOW LONG WILL I STAY IN THIS INVESTIGATION?
Children will be followed by a total of 12 months. After the child's clinical examination be conducted and depending on his/her oral health a management plan will be established. The number of times he/she must attend will depend on his oral health. The final control will be after one year. Remember that participation in this study is completely voluntary, therefore you can withdraw at any time in the study. However, we invite you to attend all appointments until the end in order to monitor the child's oral health.

WHAT IF WE WITHDRAW FROM THE INVESTIGATION?

If you decide to withdraw from the study, there will be no problem at all nor difficulties. The child will continue the oral care in this institution (without the need to be in the project). In addition, the researcher could withdraw you from the study if you do not follow his/her instructions or you do not assist to the planned appointments.

WILL I GET ANY BENEFIT FROM PARTICIPATING IN THIS INVESTIGATION?
The child will receive the benefit of being attended in the dental care in a safe way under the current pandemic situation. This management will be conducted in other countries worldwide too. This is an opportunity to learn additional aspects of oral care for the entire life.

WHAT OTHER OPTIONS DO I HAVE IN ADDITION TO PARTICIPATING IN THIS INVESTIGATION?
The option you have is not to accept to participate in the project.

HOW WILL THE PRIVACY AND CONFIDENTIALITY DATA BE GUARANTEED?
From the moment you get in the project you will be identified with a code number. Your personal data (name, ID or other information) will not be used or related to the results of the research. Data will be storage under lock. Data will be transcribed to the computer and will be maintained under security codes.
WHAT ARE THE RISKS OR DISCOMFORTS ASSOCIATED WITH THIS INVESTIGATION?
Those of a common dental care, additional risks are not involved in this study. Tooth sensitivity (pain/ discomfort associated with cold or heat). Dental care will be carried out by trained personnel for this purpose. If during the care an additional incident occurs our team will be highly trained to handle the situation.

HOW MUCH WILL I PAY TO PARTICIPATE?
Your participation in this study will be voluntary and will not have any cost.

WILL I RECEIVE ANY KIND OF COMPENSATION OR PAYMENT?
You will not receive any type of economical compensation. However, the research team will greatly appreciate your collaborating.

WHICH ARE MY RIGHTS IN THIS INVESTIGATION?
To be informed of what the study consists of and to be answered any doubts. Once the information is clear to you, you will have the right to decide whether to participate in the project and will indicate it by signing this document. You will also have the right to withdraw from the study in anytime.

WHEN WILL I FIND OUT THE FINAL DATA OF THE RESEARCH?
At the moment that you consider necessary, you can contact the researchers. They will give you the information you require. In addition, the final data of the investigation will be disclosed electronically, once all the information has been collected.

WHAT SHOULD I DO IF I HAVE ANY QUESTION OR PROBLEM?
You can contact to the research team. Their contacts are available below.

- ETHICS COMMITTEE INFORMATION
  Comité Institucional de Ética en Investigaciones, 648 9000 ext 1520, comiteetica@unbosque.edu.co, Calle 132 No. 7A-63 piso 2 y 3.

- RESEARCH GROUP INFORMATION

Stefania Martignon
Director of UNICA - Caries Research Unit
Telephone: (1) 6489000 ext. 1279
martignonstefania@unbosque.edu.co

Edgar Beltrán
UNICA - Caries Research Unit
Universidad El Bosque
Research Department
Telephone: (1) 6489000 ext. 1195-1279
ebeltranz@unbosque.edu.co

Viviana Ávila
UNICA - Caries Research Unit
Telephone: (1) 6489000 ext. 1195-1279
lavilaa@unbosque.edu.co

Part 2: Signatures.

I have been invited to participate in the study "**Caries OUT: Multicenter Study in children with CariesCare International adapted for the COVID-19 pandemic**".

I have read and understood this Informed Consent Yes ___ No ___
All my questions have been answered Yes ___ No ___
I had enough time to think about my decision Yes ___ No ___

I do not have doubts about my participation. I agree to be part of this project. When I sign this document, I will receive a copy (Parts 1 and 2).

I accept in a voluntary manner my participation and I know my right to withdraw in any moment. In addition, I know that when I sign this form, I am not renounced to any legal right.

Participant Information
Name: ___________________________Id ________________
Signature: ____________________________Date: _________________
Telephone: ________________

Witness 1 Information
Name: ___________________________Id ________________
Signature: ____________________________Date: _________________
Telephone: ________________

Witness 2 Information
Name: ___________________________Id ________________
Signature: ____________________________Date: _________________
Telephone: ________________

Principal Investigator
Name: ___________________________Id ________________
Signature: ____________________________Date: _________________
Telephone: ________________
