# Supplementary material for: CariesCare International adapted for the pandemic in children: Caries OUT multicentre single-group interventional study protocol
Source: BMC Oral Health. 2021 Jul 1;21:329. doi: 10.1186/s12903-021-01674-1 (PMC8248759; doi:10.1186/s12903-021-01674-1)
Supplement: Supplementary file 3 — Additional file 3. Written assent form. [file 12903_2021_1674_MOESM3_ESM.pdf]

UNIVERSIDAD EL BOSQUE

RESEARCH TITLE: " Caries OUT: Multicenter Study in children with CariesCare International adapted for the COVID-19 pandemic".
Informed Assent

Part 1: Research Information.

- PROTOCOL NUMBER: PCI-2019-10718
- PRINCIPAL INVESTIGATOR NAME: Edgar Beltran.
- BACKGROUND

Dental caries is a disease of teeth occurring when oral hygiene habits are deficient and the consumption of sugars is not regulated. Dental caries could be avoided if its detection and management start very early in childhood. In addition, this way we could avoid future dental pain, dental loss and chewing problems of individuals.

In this study, we propose to evaluate a new caries management system called CariesCare International or simply CCI.
The CCI management allows to identify the probability that a person will develop caries and diagnose it early based on their needs and without using rotatory instruments. Rotatory instruments work slicing damaged dental structure quickly. Its use can be replaced by the use of other instruments similar to spoons that correspond to the same function of the rotatory structures.

We are inviting you to participate in this project. Once you agree to participate CCI will be used to take care of your oral health. The results will help us to see if CCI is effective in preventing tooth decay and in maintaining good oral health. It is also very important that during the management you feel completely comfortable and satisfied with this. Therefore, at the end of the management you will be invited to answer some questions.

WHAT DOES THIS INVESTIGATION AIM?

It aims to evaluate the result of applying the CCI strategy in the management of caries in children like you from 3 to 5 years old and from 6 to 8 years old, to avoid the presence / onset of caries lesions and to identify satisfaction in the CCI management. If you agree to participate your oral health will be assessed at 4 times: at the beginning of the study, after 3, 6 and 12 months. Additional telephone controls will be conducted. In addition, based on the oral exam appropriate treatments will be performed without the need to use aerosol-generating procedures. Together, these aspects will help us to identify problems in your oral health hygiene and habits.

WHAT DO I HAVE TO DO IF I PARTICIPATE IN THIS RESEARCH?
1) Before starting the investigation: you will know about the generalities of the treatments that are handled under the CCI scheme. When you have no questions about this, you will be asked to sign this document if you agree to participate in the project, 2) During the research: you must attend to the appointments that are scheduled and follow the instructions given by the researchers (the number of appointments will depend on what is given detect in the initial oral examination) and, 3) After the investigation is finished: you might attend the follow-up of the recommendations of the investigators and guarantee and the control appointments and / or attend the follow-up telephone calls and, 4. Respond telephone satisfaction survey at the end of management.

HOW MANY PEOPLE WILL PARTICIPATE IN THIS RESEARCH?
420 children from 3 to 5 years old and/or from 6 to 8 years old.

UNIVERSIDAD EL BOSQUE Comité Institucional de Ética en Investigación

Nadia Yadira Castañeda G.

HOW LONG WILL I STAY IN THIS INVESTIGATION?

Children will be followed by a total of 12 months.

WHAT IF WE WITHDRAW FROM THE INVESTIGATION?

If you decide to withdraw from the study, there will be no problem at all nor difficulties.

WILL I GET ANY BENEFIT FROM PARTICIPATING IN THIS INVESTIGATION?

You will receive the benefit of being attended in the dental care in a safe way under the current pandemic situation.

WHAT OTHER OPTIONS DO I HAVE IN ADDITION TO PARTICIPATING IN THIS INVESTIGATION?

The option you have is not to accept to participate in the project.

HOW WILL THE PRIVACY AND CONFIDENTIALITY DATA BE GUARANTEED?

From the moment you get in the project you will be identified with a code number. Your personal data (name, ID or other information) will not be used or related to the results of the research. Data will be storage under lock and will be maintained in secret. Data will be transcribed to the computer and will be maintained under security codes.

WHAT ARE THE RISKS OR DISCOMFORTS ASSOCIATED WITH THIS INVESTIGATION?

Those of a common dental care, additional risks are not involved in this study. Tooth sensitivity (pain/ discomfort associated with cold or heat). Dental care will be carried out by trained personnel for this purpose.

HOW MUCH WILL I PAY TO PARTICIPATE?

Your participation in this study will be voluntary and will not have any cost.

WILL I RECEIVE ANY KIND OF COMPENSATION OR PAYMENT?

You will not receive any type of economical compensation. However, the research team will greatly appreciate your collaborating.

WHICH ARE MY RIGHTS IN THIS INVESTIGATION?

To be informed of what the study consists of and to be answered any doubts. Once the information is clear to you, you will have the right to decide whether to participate in the project and will indicate it by signing this document. You will also have the right to withdraw from the study in anytime.

WHEN WILL I FIND OUT THE FINAL DATA OF THE RESEARCH?

At the moment that you consider necessary, you can contact the researchers. They will give you the information you require. In addition, the final data of the investigation will be disclosed electronically, once all the information has been collected.

WHAT SHOULD I DO IF I HAVE ANY QUESTION OR PROBLEM?

You can contact to the research team. Their contacts are available below.

- ETHICS COMMITTEE INFORMATION
  Comité Institucional de Ética en Investigaciones, 648 9000 ext 1520, comiteetica@unbosque.edu.co, Calle 132 No. 7A-63 piso 2 y 3.

- RESEARCH GROUP INFORMATION

UNIVERSIDAD EL BOSQUE

Stefania Martignon
Director of UNICA - Caries Research Unit
Telephone: (1) 6489000 ext. 1279
martignonstefania@unbosque.edu.co

Edgar Beltrán

Viviana Avila
Andrea Cortés
UNICA - Caries Research Unit
Universidad El Bosque
Research Department

Part 2: Signatures.

I have been invited to participate in the study "**Caries OUT: Multicenter Study in children with CariesCare International adapted for the COVID-19 pandemic**".

I have read and understood this Informed Consent Yes ___ No ___
All my questions have been answered Yes ___ No ___
I had enough time to think about my decision Yes ___ No ___

I do not have doubts about my participation. I agree to be part of this project. When I sign this document, I will receive a copy (Parts 1 and 2).

I accept in a voluntary manner my participation and I know my right to withdraw in any moment. In addition, I know that when I sign this form, I am not renounced to any legal right.

Participant Information
Name: ___________________________ Id ________________
Signature: ____________________________ Date: _________________
Telephone: ________________

Witness 1 Information
Name: ___________________________ Id ________________
Signature: ____________________________ Date: _________________
Telephone: ________________

Witness 2 Information
Name: ___________________________ Id ________________
Signature: ____________________________ Date: _________________
Telephone: ________________

Principal Investigator
Name: ___________________________ Id ________________
Signature: ____________________________ Date: _________________
Telephone: ________________
